# Practical Notes and Formulæ

**Published:** 1886-11-20

**Authors:** 


### PRACTICAL NOTES AND FORMULÆ.

**Treatment of Diphtheria.**—Dr. J. M. Harris, Emporia, Kan., in *Medical World:* From an experience of more than forty years, I venture to give up my favorite remedy for diphtheria; the success of which is highly satisfactory, not having failed with it in a single case.

Every two hours the patient gargles with a solution of iodide of potash, a drachm to the ounce of distilled water; with chloride of sodium Labarraque's solution or bromo-chloralum alternating. Internally I give veratrum or aconite every two hours to arrest fever, and parvules of hydrag. as indicated.

Keep the room at 60°; well ventilated, and chloride of lime constantly on hand. With this treatment and proper nursing you need fear no deaths.

**Speedy Cure of Gonorrhœa.**—Dr. Chas. C. Edison, in *Chicago Medical Times:*

℞. Oil sandal wood, } aa......................ℨ iv,

Fl. ex. quillea sapo, }

M. and shake.

Add glycerin, } aa.................. ...q. s. ℥ iij.

Aqua cinnamon, }

M. Sig.—Teaspoonful four times a day.

℞. Morphia sulph..............................grs. iii,
Muriate berberina..........................grs. x,
Zinci sulphas.............................. grs. viii,
Bismuth sub nit............................ℨ iv,
Aqua rosa.................................. ℥ iv.

M. Sig.—Inject small amount after each micturation. Keep the glans penis well covered with cloth so as to prevent the discharge from soiling the linen. This is a very necessary precaution for a speedy cure, as matter upon the clothing will reinoculate and continue the disease indefinitely.

**Lemonade Iron.**—The following method of prescribing the muriated tincture of iron, as being the most palatable, elegant and altogether satisfactory method ever yet offered to the profession. Prof. Goodell, of Philadelphia, terms it "lemonade iron." I think it original with him:

℞. Mur. tinct. ferri..............................ℨ iv,

Acidi phos. dil................................ℨ vj,

Spts. limonis..................................ℨ ij,

Syr. simp. ad..................................℥ vj.

M. Sig.—Two teaspoonfuls in water after each meal. The spirits of lemon is preferred to the syrup of lemon, as the latter would render the mixture too acid. I have found the above, combined with pepsin, an excellent tonic when indications are associated with feeble digestive powers.—*Medical World.*

**Anise Cordial or Fennel Gin.**—(Used in the Infant Hospital, New York.

℞. Infusi anisi.... } ........................(℥ 2 to Oj)

Genevæ (gin).. } aa..................... : fl ℥ j.

M.—Dose, half a teaspoonful.

**Quinine in Whooping Cough.**—This is not a new reeommendation, but cumulative evidence serves to entitle it to an estimable place in our consideration. Dr. Ernest M. Lyon, of Newark, New Jersey, writes to an exchange that he has used it extensively and successfully in his practice, as follows:

℞. Quiniæ bisulph................................ʒ j.

Elix. simplic. rub., U. S. P........................℥ iij.

M. Sig.—Teaspoonful to a child aged five years and upwards. He gives it every three or four hours until cinchonism ensues, and keeps the patient under its influence, his guide for its administration being the frequency and severity of the paroxysms.—*Medical and Surgical Reporter.*

**Apone: A New Kind of Pain Expeller.**—As we imagine from its counter-irritant action, it would be very likely to relieve pain, we note that the following is said to be the mode of preparation of a sort of pain-killer recommened by Poulet (*Pharm. Zeit.*) under the name of *apone:*

℞. Capsicum................................ 20 parts,
Water of ammonia....... .............. 10 parts,
Oil of thyme........................... 1 part,
Chloral...... ......................... 1 part,
Alcohol, 60%.... ..................... 100 parts.

Digest the capsicum with the alcohol and ammonia during four weeks, filter, and add other ingredients—*Ibid.*

**Catarrhal Headache.**—Iodide of potassium is said to quickly relieve the dull headache so often accompanying an ordinary cold in the head. Two grains may be dissolved in a glassful of water, which is to be taken in little sips during half an hour. Dr. Davis recommends this simple remedy, and says he has hardly ever known it to fail.—*Ibid.*

**Glycerole for Cutaneous Pruritus.**—

℞. Acidi carbolici...........................gtts. xv,
Sodii biboratis............................grammes iii,
Glycerinæ................................ " xxx.

M. Apply with a brush over the pruriginous surfaces.—*Ibid.*

**How they do it in China.**—It is said that Dr. I. Hun Su, of Pekin, China, treats uncomplicated typhoid fever very successfully with the following prescription:

℞. Three inches dried umbilical cord,
One fried snake-skin,
One fresh tom-cat's head.

Mix. Boil in five pints of water for two hours and strain. Sig. Tablespoonful every four hours.

